# Development and Validation of Single Nucleotide Polymorphisms (SNPs) Markers from Two Transcriptome 454-Runs of Turbot (*Scophthalmus maximus*) Using High-Throughput Genotyping

**DOI:** 10.3390/ijms14035694

**Published:** 2013-03-12

**Authors:** Manuel Vera, Jose-Antonio Alvarez-Dios, Carlos Fernandez, Carmen Bouza, Roman Vilas, Paulino Martinez

**Affiliations:** 1 Laboratory of Genetics Ichthyology, Department of Biology, Faculty of Sciences, University of Girona, Campus of Montilivi s/n, Girona 17071, Spain; 2 Department of Genetics, Faculty of Veterinary, University of Santiago de Compostela, Campus of Lugo, Lugo 27002, Spain; E-Mails: carlos.fernandez.lopez@usc.es (C.F.); mcarmen.bouza@usc.es (C.B.); roman.vilas@usc.es (R.V.); paulino.martinez@usc.es (P.M.); 3 Department of Applied Mathematics, Faculty of Mathematics, University of Santiago de Compostela, Santiago de Compostela 15782, Spain; E-Mail: joseantonio.alvarez.dios@usc.es

**Keywords:** turbot, *Scophthalmus maximus*, SNP validation, EST database, non-synonymous substitution, high-throughput genotyping

## Abstract

The turbot (*Scophthalmus maximus*) is a commercially valuable flatfish and one of the most promising aquaculture species in Europe. Two transcriptome 454-pyrosequencing runs were used in order to detect Single Nucleotide Polymorphisms (SNPs) in genes related to immune response and gonad differentiation. A total of 866 true SNPs were detected in 140 different contigs representing 262,093 bp as a whole. Only one true SNP was analyzed in each contig. One hundred and thirteen SNPs out of the 140 analyzed were feasible (genotyped), while III were polymorphic in a wild population. Transition/transversion ratio (1.354) was similar to that observed in other fish studies. Unbiased gene diversity (He) estimates ranged from 0.060 to 0.510 (mean = 0.351), minimum allele frequency (MAF) from 0.030 to 0.500 (mean = 0.259) and all loci were in Hardy-Weinberg equilibrium after Bonferroni correction. A large number of SNPs (49) were located in the coding region, 33 representing synonymous and 16 non-synonymous changes. Most SNP-containing genes were related to immune response and gonad differentiation processes, and could be candidates for functional changes leading to phenotypic changes. These markers will be useful for population screening to look for adaptive variation in wild and domestic turbot.

## 1. Introduction

The turbot (*Scophthalmus maximus*; Scophthalmidae, Pleuronectiformes) is a commercially valuable flatfish that has been intensively cultured since the 1980s. Its production has steadily increased up to the present figure of 8549 tons in 2011 (91.2% European production from Spain; [1]) and it appears to be one of the most promising aquaculture species in Europe. In response to turbot industry demands, genetic markers have been developed in this species in order to evaluate genetic resources in both wild and hatchery populations and perform parentage analysis to support genetic breeding programs [2–4]. These markers have also been applied to develop genomic tools to identify genomic regions associated with productive characters [5–7] and to detect selection footprints in wild populations [8]. Increasing growth rate, controlling sex ratio (females largely outgrow males) and enhancing disease resistance currently constitute the main goals of genetic breeding programs in this species.

The necessity of understanding the immune response to pathogens of industrial relevance and to identify genes involved in the sex differentiation pathway led us to increase genomic resources in turbot. As a consequence thereof, an Expressed Sequence Tag (EST) database from cDNA libraries of the main immune tissues was constructed using Sanger sequencing [9]. Recently, this database has been amplified with two 454 FLX runs [10,11] (454-Life Sciences, Brandford, CT, USA; for 454-technique methodology see [12,13]). Next Generation Sequencing (NGS) technologies offer the ability to produce an enormous volume of data with a very low sequencing cost per base [12]. Thus, this turbot EST database is currently composed of ~70,000 unique sequences (~20,000 contigs and ~50,000 singletons). ESTs are essential to ascertain the gene [14,15], but also to identify polymorphic gene-associated markers, such as microsatellites and single nucleotide polymorphisms (SNPs) (type I markers; [9,16–18]). Type I markers are very useful for constructing genetic or physical maps, and for comparative mapping [7,19,20].

SNPs have several advantages over other markers when it comes to mapping genes or inferring population structure [21]. They can be easily evaluated *in silico* off public databases and their genotypes quickly assessed by mini-sequencing reactions [9,22] or by high-throughput technologies [23,24]. SNP alleles are almost exclusively identical-by-descent (IBD) and thus they prevent scoring errors associated to homoplasy [25]. They are extremely stable, due to low mutation rates [26], and occur more often in the genome than other markers. In the human genome, for instance, there is on average 1 SNP per 300 bp [27], and their frequency in non-model species has been estimated at ~1 in 200–500 bases for non-coding DNA and ~1 in 500–1000 bases for coding DNA [28]. In turbot, Vera *et al.* [29] estimated 1 true SNP every ~100 bp from the EST database composed only of Sanger sequences, suggesting the existence of large SNP resources in this species. During the last decade, SNP discovery pipelines have been developed for aquaculture species including fish [18,30–35], shellfish [36–38] and crustaceans [39,40]. In turbot, a SNP calling tool was included in the turbot database [9] and it has been refined in the updated version [11]. In this study, we screened genomic resources available in an updated version of the turbot EST database using contigs containing NGS 454-sequences to identify and characterize SNPs associated to immune- and reproduction-related genes. These markers will be used for further structural genomic analysis focused on quantitative trait loci (QTLs) linked to productive traits, as well as for population screening to look for adaptive variation in wild and domestic turbot.

## 2. Results and Discussion

### 2.1. Database Exploitation and SNP Detection

The main characteristics of the turbot 454-transcriptome sequencing runs have been described in previous studies [10,11]. The used database (version 4.0 September 2011) was constituted by 71,033 unique sequences, 18,880 contigs and 52,153 singletons including 454-sequences and Sanger sequences [9] with a total length of 52,402,177 base pairs (bp, ~52 Mb). However, in order to avoid duplicates with the previous SNPs developed from sequences obtained with Sanger methodology [29], and since we were mainly interested in validating SNPs at new immune- and reproduction-related genes, only contigs composed exclusively of at least four 454-sequences were used for SNP detection. Thus, 140 contigs from the turbot database, which met these requirements, were taken into account for the SNP development. The total length analyzed was 262,093 bp and contig length ranged from 728 bp to 4885 bp, with a mean length value of 1872.09 ± 746.69 bp. The total number of true SNPs detected using the program QualitySNP (for true SNP definition see the experimental section) was 866, SNP number per contig ranged from 1 to 58, with a mean value of 6.18 ± 8.34. Thus, the expected frequency of SNP appearance in the analyzed sequences would be 1 SNP every 302 bp. This value is lower than that previously reported in *S. maximus* (1 SNP each ~100 bp; [29]), but similar to those described in non-model species [28]. The success of any genotyping method is reflected in what is referred to as the conversion rate and the global success rate. The former only considers the polymorphic markers, whereas the latter considers all the markers (monomorphic and polymorphic) that were successfully typed within the analyzed samples [41]. Of the 140 true SNPs tested, 27 (19.3%) could not be genotyped, and thus they were considered to be genotyping failures due to technical and/or genotyping problems. Only 2 out of the 113 feasible SNPs (see definition in the experimental section) were monomorphic. Therefore, the global success rate and conversion rate were 80.7% and 79.3%, respectively. Global success rate was very similar to that previously described in the species (78.4%), but conversion rate was much higher than previously reported using sequences from cDNA libraries (37.7%; see Vera *et al.* [29]), likely due to the different library construction methods and bioinformatic pipeline approaches followed in 454 and Sanger contigs (see experimental section).

### 2.2. SNP Performance

**Figure 1:**
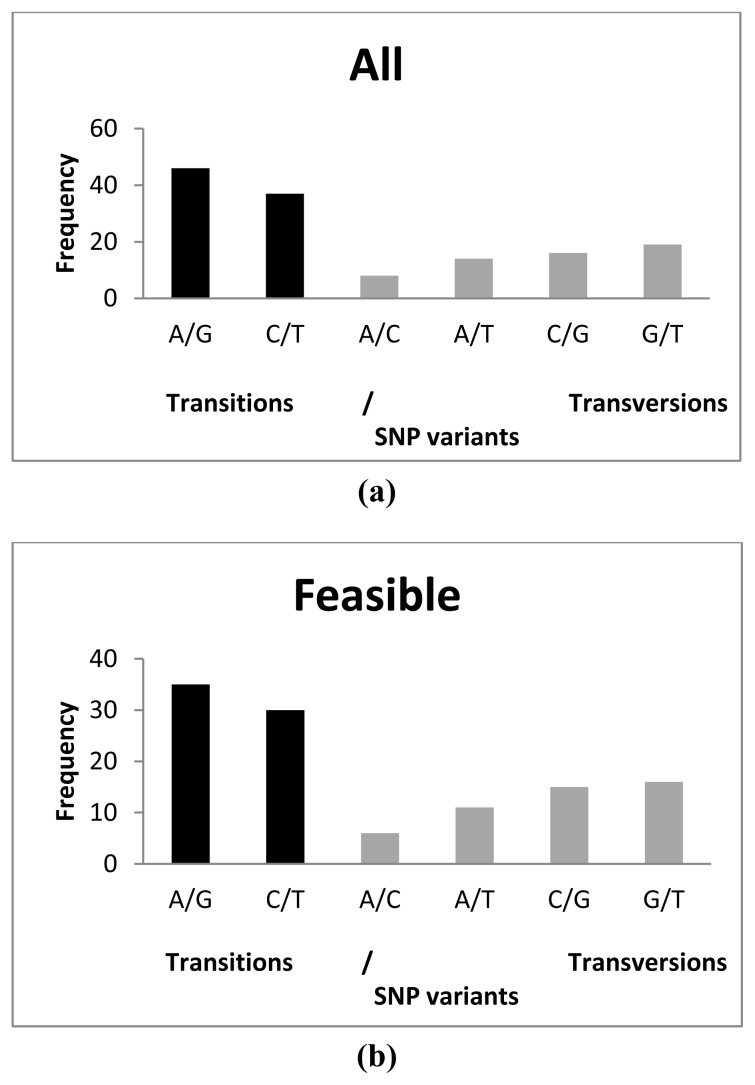
Distribution of SNP variants analyzed in this study (**a**) using all SNPs tested; (**b**) using only feasible SNPs. Transitions (ts) and transversions (tv) are indicated in black and grey colour, respectively.

A total of 65 transitions (A/G and C/T) and 48 transversions (A/C, A/T, C/G and G/T) were detected among feasible SNPs, A/G being the most common (35) and A/C the least common (6) substitutions observed (Figure 1). This represented a transition/transversion (ts/tv) ratio of 1.354. This ratio was lower than that observed by Vera *et al.* [29] (1.885) and *in silico* (1.456) by Pardo *et al.* [9], but it was very similar to that described for common carp (*Cyprinus carpio*) (1.310) [42] and gilthead seabream (*Sparus aurata*) (1.375) [31]. Also, the most frequent transitions and transversions differed from previous reports: C/T and G/T, respectively [29], and A/G and A/C [9]. These discrepancies could be due to the opposite sequencing directions, as all sequences by Vera *et al.* [29] and Pardo *et al.* [9] were obtained from the 3′ end using cDNA libraries, while those from the 454-run were randomly obtained by fragmentation of the whole cDNA according to the cDNA rapid library preparation method (Roche Farma, S. A. [43]). Moreover, the longer coding region portion analyzed in 454-runs regarding Sanger sequencing in our study may determine differences because of the different selective constraints of UTR regarding coding regions. No differences were detected among distribution of the variants between tested SNPs and feasible SNPs (χ^2^ = 0.3115; *p* = 0.9974). All polymorphic SNP loci showed two alleles and all of them agreed with those expected from the database information.

### 2.3. SNP Diversity

Only two loci among the 113 feasible SNPs were monomorphic (SmaSNP_287 and SmaSNP_334). Among polymorphic SNPs, unbiased gene diversity (He) estimates ranged from 0.060 at SmaSNP_237, SmaSNP_245 and SmaSNP_305 to 0.510 at SmaSNP_225 with a mean value of 0.344 ± 0.149. The minimum allele frequency (MAF) in the polymorphic markers ranged from 0.030 (SmaSNP_237, SmaSNP_245 and SmaSNP_305) to 0.500 in SmaSNP_249 with a mean value of 0.259 ± 0.140. Departures from Hardy-Weinberg equilibrium (HWE) were detected in five markers (SmaSNP_253, SmaSNP_271, SmaSNP279, SmaSNP_289, SmaSNP_326; Table 1), although all markers were at equilibrium after Bonferroni correction (*p* = 0.0004). The samples from the Cantabrian turbot population were globally in accordance with HWE expectations when tested simultaneously for all loci (*p* = 0.9999). These polymorphic values were in the range to those previously described in the species [29], and they were also similar to those reported in other fish species [42,44]. No Linkage disequilibrium (LD) was detected among the 6328 loci pairs after Bonferroni correction (*p* = 0.0004).

### 2.4. SNP Position within Genes: Synonymous *vs*. Non-Synonymous Substitutions

Consensus sequences of contigs containing polymorphic SNPs were compared using NCBI BLAST with public databases, namely UniRef90, NCBI’s nr, KEGG, COG, PFAM, LSU and SSU. The subsequent BLAST output was then parsed with Auto FACT [45]. All contigs containing feasible SNPs were annotated (except SmaSNP_320, Table 1). The informative strand, reading frame, and stop codon at each contig were recorded using homology with the highest homologous annotated sequence in public databases. Nine feasible SNPs (8.0%) could not be positioned, because no consistent reading frames were detected (indicated as “unknown” location on Table 2). Fifty-five SNPs (48.7%) were located in untranslated regions (UTR), either in the 5′ UTR (17, 15.0%) or 3′ UTR (38, 33.6%), which is in accordance with the approximately double length of 3′ compared to 5′ UTR [9]. On the other hand, 49 SNPs (43.4%) were localized in the coding region (Table 2), a percentage of SNPs higher than previously reported in the species (24.7%, [29]) and in other aquaculture fish species (e.g., Atlantic salmon 24%, [32]; Atlantic cod 17.4%, [34]). All these studies followed a 3′ UTR Sanger sequencing strategy, and therefore the coding region was less represented than in the case of the 454 Roche runs after a cDNA rapid library preparation protocol, which accounts for the differences observed. This result shows the utility of the NGS methodologies for SNP detection in the coding region. Thirty-three (29.2%) of these 49 SNPs were synonymous, and 16 (14.2%) were non-synonymous. On the other hand, the relationship between synonymous *vs.* non-synonymous changes (2:1) was lower than in other species [46,47]. Evolutionary constraints should preferentially eliminate non-synonymous variation because it is usually associated with deleterious mutations [35].

Non-synonymous SNPs in coding regions represent alternative allelic variants of a gene, which can determine functional changes in the corresponding proteins and lead to phenotypic changes. Among these genes there can be found a retinol dehydrogenase (SmaSNP_264), three zona pellucida proteins (SmaSNP_212, SmaSNP_217, SmaSNP_282) related to reproduction processes, and a lipocalin (SmaSNP_325) involved in tear secretion (Table 2).

In the present study, we used sequences obtained from two transcriptome 454-pyrosequencing runs, one related to immune system [10] and another one from the hypothalamic pituitary-gonad axis [11]. Thus, GO terms were mainly related to immune response and reproduction processes (Table 2). The non-synonymous variation was associated with genes involving either immune response or sex differentiation processes. A large number of SNP linked to annotated genes were identified and validated. This set of markers are being used for population genomic studies and turbot genetic map enrichment, both approaches providing useful information for evolutionary and turbot industry applied studies.

## 3. Experimental Section

### 3.1. EST Database, SNP Detection and Annotation

Sequences were obtained from two transcriptome 454-pyrosequencing runs of turbot cDNA libraries, one belonging to the immune transcriptome [10] and another one from the hypothalamic pituitary-gonad axis [11]. A brief description of both runs is shown in Table 3. All the 454-reads were assembled with MIRA [48], and they make up the 454-sequences incorporated into the turbot database. In order to create contigs and detect SNPs, these 454-sequences were assembled alongside Sanger sequences available [9] in the database with CAP3 [49] using default parameters. This is a common strategy when dealing with hybrid Sanger-454 assemblies [50]. The resulting ACE format assembly file was fed into QualitySNP [51] in conformity with the bioinformatic pipeline described by Vera *et al.* [29]. Briefly, QualitySNP uses three filters for the identification of reliable SNPs: Filter 1 screens for all potential SNPs with the requirement that every allele is represented in more than one sequence (each contig has to have at least a depth of 4 sequences); filter 2 uses a haplotype-based strategy to detect reliable SNPs after reconstructing confident haplotypes with an algorithm that minimizes false haplotypes due to the occurrence of sequencing errors; and filter three screen SNPs by calculating a confidence score based on sequence redundancy and quality (only sequences with PHRED >20 were used). SNPs that pass filters 1 and 2 are called real SNPs and those passing all filters are called true SNPs [51]. Resulting files were processed with our own custom Perl programs to extract relevant information. The obtained data were imported into a mySQL server [52]. A user-friendly web access interface was designed so that contig graphs are clickable and the output visually refined with color-coded nucleotide views [53]. A graphical interface allowing for SNP database search by alleles, contig depth, and annotation was set up. EST annotation of these contigs was performed using BLASTx, which searches proteins using a translated nucleotide query [54]. Only E-values lower than 10^−5^ were considered for gene annotation (Table 1, Table S1).

### 3.2. SNP Genotyping and Validation

DNA of all individuals analyzed was extracted from a piece of caudal fin using standard phenol-chloroform procedures [55].

SNPs identified were validated and genotyped with the MassARRAY platform (Sequenom, San Diego, CA, USA) following the protocols and recommendations provided by the manufacturer. Briefly, the technique consists of an initial locus-specific polymerase chain reaction (PCR), followed by single-base extension using mass-modified dideoxynucleotide terminators of an oligonucleotide primer that anneals immediately upstream of the polymorphic site (SNP) of interest (see [56,57] for more technical information). The distinct mass of the extended primer identifies the SNP allele. Primer sequences, SNP position, expected variants and annotation for the 140 tested SNPs are shown on Supplementary Table 1. MALDI-TOF mass spectrometry analysis in an Autoflex spectrometer was used for allele scoring.

Assays were designed for 140 true SNPs always located in different sequences and were combined in 7 multiplex reactions including 24 SNPs each except for multiplex 5 (23 SNPs), 6 (18 SNPs) and 7 (3 SNPs) (see Supplementary Table 1 for multiplex information). SNP multiplexes were designed *in silico* and tested on a panel of 8 turbot individuals from a wild Cantabrian (northern Spain) population. SNPs were classified based on manual inspection as “failed assays” (in case that the majority of genotypes could not be scored and/or the samples did not cluster well according to genotype), and feasible SNPs (markers with proper and reliable genotypes), these being either monomorphic or polymorphic.

### 3.3. Gene Diversity and Population Analysis

In order to estimate genetic diversity parameters, all SNPs were genotyped for polymorphism evaluation in a sample of 33 individuals (including the 8 individuals used for marker performance) from the wild Cantabrian population previously used.

Estimates of genetic diversity (unbiased expected heterozygosity (He) and minimum allele frequency (MAF)) were estimated using FSTAT 2.9.3 [58]. The conformance to Hardy-Weinberg (HW) and genotypic equilibria were obtained using GENEPOP 4.0 [59,60]. Conformance to HWE was checked using the complete enumeration method [61] because only two alleles were detected at each locus. Bonferroni correction was applied when multiple tests were performed [62].

### 3.4. Detection of Synonymous/Non-Synonymous SNPs

All the six possible reading frames of the consensus sequence of each containing SNP functionally annotated contig were obtained using ORF (Open Reading Frame) Finder application [63]. The best candidate frame (usually the longest one) was compared against the NCBI protein database using BLASTp and BLASTx, and the protein with highest E-value was downloaded and aligned with the selected frame for SNP location using Clustal W [64] implemented in BioEdit v. 7.1. [65]. This approach enabled us to locate SNPs by looking at the coding region. For those SNPs in the coding region, the resulting amino acid sequences of both variants were translated to determine whether SNP variants were synonymous or non-synonymous. Gene onthology (GO) terms were searched using QUICKGO [66] and AmiGO [67] utilities.

## 4. Conclusions

A total of 140 contigs (total length 262,093 bp) formed exclusively by 454-pyrosequencing reads were used to identify new putative SNPs in *S. maximus*. One hundred and thirteen SNPs of the 140 tested were amplified and genotyped, 111 being polymorphic in a wild Cantabrian population, showing the utility of the new NGS techniques for true SNP detection (conversion rate = 79.3%). Diversity levels at the population were similar to previous studies [29,42,44] and were in accordance with HWE expectations. An important number of these polymorphic SNPs were located in the coding region and 16 of them (14.4%) represented non-synonymous changes at genes related to immune response and gonad differentiation processes as shown by the detected GO terms. Therefore, these SNPs are valuable resources for future population genetics, high-resolution genetic maps, quantitative trait loci (QTL) identification, association studies and marker assisted selection (MAS) breeding in turbot.

## Figures and Tables

**Table 1:** Annotation, variants and diversity values of the 113 technically feasible SNPs in the Cantabric turbot population (33 individuals) used in this study.

SNP Name	Annotation	Variants	MAF	P (HW)	He	Fis
SmaSNP_211	Cyclin-dependent kinase 2 interacting protein	A/T	A = 0.152	0.1307	0.262	0.307
SmaSNP_212	Zona pellucida sperm-binding protein 3	A/G	A = 0.152	0.4198	0.265	0.179
SmaSNP_215	Mitotic specific cyclin-B1	C/T	T = 0.212	0.2948	0.338	−0.255
SmaSNP_216	Pre-mRNA branch site protein p14	A/T	T = 0.348	0.7003	0.460	−0.119
SmaSNP_217	Zona pellucida protein C1	A/G	G = 0.258	0.1616	0.390	0.301
SmaSNP_218	Mitochondrial ribosomal protein S18A	G/T	T = 0.333	1.0000	0.452	0.061
SmaSNP_219	U3 small nucleolar ribonucleoprotein protein IMP3	G/T	G = 0.409	1.0000	0.491	−0.050
SmaSNP_220	Coatomer subunit epsilon isoform 1	C/T	T = 0.197	0.5750	0.322	0.153
SmaSNP_222	Signal recognition particle 14 kDa protein	G/T	G = 0.203	1.0000	0.329	−0.046
SmaSNP_223	Epithelial cell adhesion protein	A/T	T = 0.333	1.0000	0.452	0.061
SmaSNP_224	Transcription initiation factor TFIID subunit D11	C/G	G = 0.182	1.0000	0.302	−0.003
SmaSNP_225	Acidic ribosomal protein P1	A/G	G = 0.480	1.0000	0.510	0.059
SmaSNP_226	Alcohol dehydrogenase Class-3	C/T	T = 0.288	0.6913	0.416	−0.093
SmaSNP_227	Thioredoxin protein 4A	A/G	A = 0.242	1.0000	0.373	0.025
SmaSNP_228	Novel protein similar to vertebrate THAP domain containing 4 (THAP4)	A/G	G = 0.212	0.6068	0.340	0.109
SmaSNP_229	Tumor suppressor candidate 2	A/G	A = 0.031	1.0000	0.061	−0.016
SmaSNP_230	Optic atrophy 3 protein	C/T	T = 0.266	0.6477	0.397	0.135
SmaSNP_231	RNA 3′-terminal phosphate cyclase	A/C	C = 0.266	0.6475	0.397	0.135
SmaSNP_232	RAD1 homolog	A/G	A = 0.438	0.4921	0.501	0.127
SmaSNP_233	Ubiquitin carrier protein	G/T	T = 0.409	1.0000	0.491	−0.050
SmaSNP_234	chromatin accessibility complex protein 1	A/G	G = 0.047	0.0504	0.092	0.659
SmaSNP_235	Nucleolar protein 16	A/G	G = 0.258	0.4023	0.389	0.144
SmaSNP_236	Isopentenyl-diphosphate delta-isomerase 1	C/G	G = 0.141	0.4763	0.246	0.111
SmaSNP_237	Ran-specific GTPase-activating protein	G/T	T = 0.030	1.0000	0.060	−0.016
SmaSNP_238	Forkhead box H1	A/G	A = 0.453	1.0000	0.503	−0.056
SmaSNP_239	Stathmin	C/T	C = 0.258	0.6436	0.387	−0.174
SmaSNP_240	Ubiquinol-cytochrome c reductase core I protein	C/T	C = 0.152	0.5521	0.261	0.072
SmaSNP_241	BolA-like protein 3	C/G	G = 0.313	0.4371	0.438	0.143
SmaSNP_243	ce ceroid-lipofuscinosis neuronal protein 5	G/T	G = 0.455	1.0000	0.504	0.038
SmaSNP_244	SSU rRNA; *Psetta* maxima (turbot)	C/T	C = 0.061	1.0000	0.116	−0.049
SmaSNP_245	Chromobox protein homolog 3	G/T	T = 0.030	1.0000	0.060	−0.016
SmaSNP_246	Transmembrane protein 208	A/C	A = 0.469	0.7198	0.505	−0.114
SmaSNP_247	Ribosomal protein L18a	A/C	A = 0.234	1.0000	0.365	0.058
SmaSNP_248	Pre-mRNA-processing factor 19	C/T	C = 0.318	1.0000	0.440	−0.032
SmaSNP_249	Alpha-l-fucosidase	A/G	A = 0.500	0.7275	0.509	0.106
SmaSNP_250	Protein phosphatase 2 (Formerly 2A)	A/G	G = 0.406	0.0598	0.493	0.366
SmaSNP_252	LON peptidase *N*-terminal domain and RING finger protein 1	G/T	T = 0.167	1.0000	0.282	0.034
SmaSNP_253	IK cytokine	A/G	A = 0.439	0.0348	0.497	−0.402
SmaSNP_256	Ribonuclease UK114	C/T	C = 0.232	0.6038	0.364	0.116
SmaSNP_257	Inner centromere protein	A/G	G = 0.303	0.4239	0.430	0.154
SmaSNP_259	Beta-galactoside-binding lectin	C/T	C = 0.379	0.7242	0.477	−0.079
SmaSNP_260	Enoyl-Coenzyme A hydratase	A/T	A = 0.273	0.3819	0.402	−0.208
SmaSNP_261	Sept2 protein	A/G	G = 0.197	1.0000	0.321	−0.038
SmaSNP_262	DNA-directed RNA polymerase I subunit RPA34	A/G	A = 0.078	1.0000	0.146	−0.069
SmaSNP_263	Epithelial membrane protein 2	A/G	G = 0.379	0.1336	0.480	0.306
SmaSNP_264	Retinol dehydrogenase 3	C/G	G = 0.409	1.0000	0.491	−0.050
SmaSNP_265	WD repeat-containing protein 54	A/G	A = 0.076	1.0000	0.142	−0.067
SmaSNP_266	tRNA pseudouridine synthase 3	C/T	C = 0.136	0.4637	0.240	0.115
SmaSNP_267	Transmembrane protein 167 precursor	G/T	G = 0.258	0.6463	0.387	−0.174
SmaSNP_270	Flotillin-1	C/G	G = 0.438	0.1694	0.502	0.253
SmaSNP_271	NAD(P)H dehydrogenase quinone 1	A/G	G = 0.359	0.0488	0.471	0.403
SmaSNP_273	Ubiquitin protein ligase E3 component	C/T	C = 0.484	0.7353	0.508	0.077
SmaSNP_274	K13213 matrin 3	C/G	G = 0.106	0.2983	0.193	0.216
SmaSNP_275	Dolichol-phosphate mannosyltransferase	A/G	A = 0.091	0.2209	0.169	0.281
SmaSNP_276	DNA-directed RNA polymerases i II and III subunit rpabc1	A/G	A = 0.197	0.5728	0.322	0.153
SmaSNP_277	Syndecan 2	A/C	A = 0.429	1.0000	0.505	−0.130
SmaSNP_278	Peptide methionine sulfoxide reductase	C/G	C = 0.078	1.0000	0.146	−0.069
SmaSNP_279	Methyltransferase-like protein 21D	G/T	G = 0.470	0.0129	0.509	0.465
SmaSNP_281	Phosphatidylinositol transfer protein beta isoform-like isoform 2	C/T	T = 0.318	0.4333	0.439	−0.172
SmaSNP_282	Zona pellucida protein C	A/T	T = 0.121	1.0000	0.216	−0.123
SmaSNP_283	AP-2 complex subunit alpha-2-like	G/T	G = 0.333	0.2669	0.450	−0.213
SmaSNP_284	Apoptosis regulator BAX	A/G	G = 0.409	0.0780	0.493	0.324
SmaSNP_285	Borealin	G/T	T = 0.032	1.0000	0.063	−0.017
SmaSNP_286	Brain protein 44	C/T	C = 0.394	0.2691	0.483	−0.255
SmaSNP_287	Exosome component 8	A/G	G =1.000	-	0.000	NA
SmaSNP_288	Atrophin-1 domain containing protein	G/T	T = 0.439	1.0000	0.500	−0.030
SmaSNP_289	similar to connectin/titin	A/T	A = 0.303	0.0018	0.433	0.580
SmaSNP_290	Ubiquitin carboxyl-terminal hydrolase L5	C/T	T = 0.469	1.0000	0.506	0.012
SmaSNP_292	Histone deacetylase complex subunit SAP18	C/T	C = 0.188	0.5568	0.308	−0.216
SmaSNP_293	Replication protein A 14 kDa subunit	C/G	G = 0.182	1.0000	0.302	−0.003
SmaSNP_296	Carbonic anhydrase	G/T	G = 0.076	1.0000	0.142	−0.067
SmaSNP_297	UPF0414 transmembrane protein	C/T	C = 0.212	0.6080	0.340	0.109
SmaSNP_298	Queuine tRNA-ribosyltransferase	C/T	T = 0.061	1.0000	0.116	−0.049
SmaSNP_299	NHP2-like protein 1	C/G	C = 0.379	0.1358	0.480	0.306
SmaSNP_304	Microsomal glutathione *S*-transferase 3	A/G	A = 0.091	1.0000	0.168	−0.085
SmaSNP_305	Actin related protein 2/3 complex subunit 4	C/T	T = 0.030	1.0000	0.060	−0.016
SmaSNP_306	Cyclophilin B	C/G	C = 0.061	1.0000	0.116	−0.049
SmaSNP_307	Dynein light chain Tctex-type 3	C/G	C = 0.061	1.0000	0.116	−0.049
SmaSNP_308	Ependymin-1	A/G	A = 0.234	0.3135	0.366	0.231
SmaSNP_309	C-4 methylsterol oxidase	A/G	A = 0.297	1.0000	0.424	0.043
SmaSNP_310	Dynein light chain LC8-type	G/T	T = 0.045	1.0000	0.088	−0.032
SmaSNP_311	Rho-related GTP-binding protein RhoF	A/T	T = 0.394	0.2669	0.483	−0.255
SmaSNP_312	Golgi SNAP receptor complex member 1	A/T	A = 0.188	0.5587	0.308	−0.216
SmaSNP_314	Ribosomal L1 domain-containing protein 1	A/G	A = 0.203	1.0000	0.329	−0.046
SmaSNP_315	*N*-alpha-acetyltransferase 50	A/T	A = 0.242	1.0000	0.373	0.025
SmaSNP_316	Oncogene DJ-1 isoform 1	C/T	C = 0.453	1.0000	0.503	−0.056
SmaSNP_317	Wu:fj40d12 protein *n* = 7 Tax = Euteleostomi RepID = A3KP21_DANRE	A/G	A = 0.438	1.0000	0.500	0.000
SmaSNP_318	Mucin multi-domain protein	C/G	C = 0.167	0.5617	0.281	−0.185
SmaSNP_319	Adenosine kinase	A/G	A = 0.182	0.5575	0.301	−0.208
SmaSNP_320	No homology found	A/G	A = 0.394	0.4901	0.486	0.127
SmaSNP_321	Zymogen granule membrane protein 16	A/G	G = 0.333	1.0000	0.451	−0.076
SmaSNP_322	6-Pyruvoyl tetrahydrobiopterin synthase	C/T	C = 0.031	1.0000	0.061	−0.016
SmaSNP_323	Proteasome subunit beta	C/T	T = 0.125	1.0000	0.222	−0.127
SmaSNP_324	RING finger protein 4	A/G	A = 0.394	0.0652	0.488	0.379
SmaSNP_325	Lipocalin	C/G	C = 0.136	1.0000	0.239	−0.143
SmaSNP_326	Choline transporter-like protein 2	A/G	G = 0.455	0.0311	0.507	0.402
SmaSNP_328	RNA-binding proteins (RRM domain)	C/T	C = 0.106	1.0000	0.192	−0.103
SmaSNP_329	Type II keratin	C/G	G = 0.061	1.0000	0.116	−0.049
SmaSNP_330	Novel protein similar to vertebrate thyroid hormone receptor interactor 12 (TRIP12)	C/T	T = 0.094	1.0000	0.172	−0.088
SmaSNP_332	Ribosomal protein S6 kinase	A/C	A = 0.470	0.7287	0.507	0.103
SmaSNP_333	Transmembrane 6 superfamily member 2	A/T	T = 0.288	0.0796	0.419	0.348
SmaSNP_334	PREDICTED: hypothetical protein LOC100712283 [Oreochromis niloticus]	C/T	T =1.000	-	0.000	NA
SmaSNP_337	1-Alkyl-2-acetylglycerophosphocholine esterase	C/T	C = 0.234	1.0000	0.365	0.058
SmaSNP_338	CD151 antigen	C/T	T = 0.266	0.3909	0.395	−0.186
SmaSNP_339	Arsenite methyltransferase 1	A/T	A = 0.313	1.0000	0.436	−0.002
SmaSNP_340	Receptor expression-enhancing protein 5	C/T	T = 0.234	0.6507	0.364	−0.116
SmaSNP_341	Cathepsin *S*	C/G	G = 0.333	0.1119	0.454	0.332
SmaSNP_342	Trans-1,2-dihydrobenzene-1,2-diol dehydrogenase	A/G	A = 0.424	0.2818	0.494	−0.226
SmaSNP_343	High mobility group protein 2	G/T	G = 0.470	0.2980	0.508	0.224
SmaSNP_346	ATP-binding cassette, sub-family A (ABC1)	C/T	T = 0.288	1.0000	0.417	0.055
SmaSNP_347	Myomesin 1a (skelemin)	C/T	T = 0.091	1.0000	0.168	−0.085
SmaSNP_348	Retinoic acid receptor responder protein 3	A/G	G = 0.439	1.0000	0.500	−0.030
SmaSNP_349	Nucleophosmin 1	A/C	A = 0.258	0.6466	0.387	−0.174

**Table 2:** Predicted position, SNP location within genes and their correspondent synonymous *vs.* non-synonymous variants of the 113 technically feasible SNPs.

SNP Name	SNP location/effect	GO term
SmaSNP_211	3′ UTR	phosphorylation (GO:0016310)
SmaSNP_212	Non synonymous	reproduction (GO:0000003)
SmaSNP_215	Synonymous	mitotic cell cycle (GO:0000278)
SmaSNP_216	3′ UTR	protein localization to cell division site (GO:0072741)
SmaSNP_217	Non synonymous	binding of sperm to zona pellucida ( GO:0007339)
SmaSNP_218	Non synonymous	protein import into mitochondrial matrix (GO:0030150)
SmaSNP_219	3′ UTR	ribonucleoprotein complex biogenesis (GO:0022613)
SmaSNP_220	Synonymous	ribosomal large subunit assembly (GO:0000027)
SmaSNP_222	5′ UTR	regulation of peptidoglycan recognition protein signaling pathway (GO:0061058)
SmaSNP_223	Synonymous	cell adhesion (GO:0007155)
SmaSNP_224	Synonymous	DNA-dependent transcription, initiation (GO:0006352)
SmaSNP_225	3′ UTR	ribosomal large subunit assembly (GO:0000027)
SmaSNP_226	Synonymous	cellular alcohol metabolic process (GO:0044107)
SmaSNP_227	Synonymous	thioredoxin biosynthetic process (GO:0042964)
SmaSNP_228	5′ UTR	regulation of nucleotide-binding oligomerization domain containing signaling pathway (GO:0070424 )
SmaSNP_229	3′ UTR	immune response to tumor cell (GO:0002418)
SmaSNP_230	3′ UTR	reproduction (GO:0000003)
SmaSNP_231	Non synonymous	phosphorylation of RNA polymerase II *C*-terminal domain (GO:0070816)
SmaSNP_232	Synonymous	resolution of meiotic recombination intermediates (GO:0000712)
SmaSNP_233	Synonymous	ubiquitin-dependent protein catabolic process (GO:0006511)
SmaSNP_234	3′ UTR	regulation of macrophage inflammatory protein 1 alpha production (GO:0071640)
SmaSNP_235	Synonymous	protein localization to nucleolar rDNA repeats (GO:0034503)
SmaSNP_236	Synonymous	T-helper 1 cell activation (GO:0035711)
SmaSNP_237	5′ UTR	termination of G-protein coupled receptor signaling pathway (GO:0038032)
SmaSNP_238	Non synonymous	transcription initiation from RNA polymerase III type 2 promoter (GO:0001023)
SmaSNP_239	5′ UTR	Not found
SmaSNP_240	Synonymous	MHC class I protein complex assembly (GO:0002397)
SmaSNP_241	3′ UTR	reproduction (GO:0000003)
SmaSNP_243	Non synonymous	neuronal stem cell maintenance (GO:0097150)
SmaSNP_244	Unknown	Not found
SmaSNP_245	5′ UTR	reproduction (GO:0000003)
SmaSNP_246	Synonymous	intracellular protein transmembrane transport (GO:0065002)
SmaSNP_247	Non synonymous	ribosomal protein import into nucleus (GO:0006610)
SmaSNP_248	3′ UTR	regulation of mitotic recombination (0000019)
SmaSNP_249	3′ UTR	alpha-l-fucosidase activity (GO:0004560)
SmaSNP_250	3′ UTR	modulation by virus of host protein serine/threonine phosphatase activity (GO:0039517)
SmaSNP_252	3′ UTR	regulation of macrophage inflammatory protein 1 alpha production (GO:0071640)
SmaSNP_253	Synonymous	regulation of cytokinesis (GO:0032465)
SmaSNP_256	Synonymous	regulation of ribonuclease activity (GO:0060700)
SmaSNP_257	Synonymous	centromere complex assembly (GO:0034508)
SmaSNP_259	3′ UTR	complement activation, lectin pathway (GO:0001867)
SmaSNP_260	3′ UTR	amitosis (GO:0051337)
SmaSNP_261	3′ UTR	protein processing (GO:0016485)
SmaSNP_262	Synonymous	RNA polymerase I transcriptional preinitiation complex assembly (GO:0001188)
SmaSNP_263	Synonymous	membrane protein proteolysis (GO:0033619)
SmaSNP_264	Non synonymous	reproduction (GO:0000003)
SmaSNP_265	5′ UTR	ribosomal subunit export from nucleus (GO:0000054)
SmaSNP_266	Synonymous	reproduction (GO:0000003)
SmaSNP_267	3′ UTR	smoothened signaling pathway involved in regulation of cerebellar granule cell precursor cell proliferation (GO:0021938)
SmaSNP_270	3′ UTR	flotillin complex (GO:0016600)
SmaSNP_271	Synonymous	NAD(P)H dehydrogenase complex assembly (GO:0010275)
SmaSNP_273	3′ UTR	regulation of ubiquitin-protein ligase activity involved in mitotic cell cycle (GO:0051439)
SmaSNP_274	Unknown	reproduction (GO:0000003)
SmaSNP_275	Synonymous	dolichyl-phosphate beta-d-mannosyltransferase activity (GO:0004582)
SmaSNP_276	Synonymous	transcription from RNA polymerase III type 2 promoter (GO:0001009)
SmaSNP_277	3′ UTR	T-helper 2 cell activation (GO:0035712)
SmaSNP_278	3′ UTR	cellular response to methionine (GO:0061431)
SmaSNP_279	Non synonymous	protein import (GO:0017038)
SmaSNP_281	5′ UTR	regulation of beta 2 integrin biosynthetic process (GO:0045115)
SmaSNP_282	Non synonymous	regulation of binding of sperm to zona pellucida (GO:2000359)
SmaSNP_283	3′ UTR	cellular macromolecular complex subunit organization (GO:0034621)
SmaSNP_284	Non synonymous	regulation of apoptotic process (GO:0042981)
SmaSNP_285	Synonymous	chromosome passenger complex localization to kinetochore (GO:0072356)
SmaSNP_286	5′ UTR	brain development (GO:0007420)
SmaSNP_287	Non synonymous	extracellular vesicular exosome assembly (GO:0071971)
SmaSNP_288	Unknown	Not found
SmaSNP_289	Unknown	Not found
SmaSNP_290	Synonymous	regulation of ubiquitin-specific protease activity (GO:2000152)
SmaSNP_292	3′ UTR	suppression by virus of host TAP complex (GO:0039589)
SmaSNP_293	3′ UTR	DNA replication preinitiation complex assembly (GO:0071163)
SmaSNP_296	Synonymous	carbon utilization (GO:0015976)
SmaSNP_297	3′ UTR	membrane protein proteolysis (GO:0033619)
SmaSNP_298	Non synonymous	queuine tRNA-ribosyltransferase activity (GO:0008479)
SmaSNP_299	Synonymous	Not found
SmaSNP_304	Synonymous	reproduction (GO:0000003)
SmaSNP_305	3′ UTR	protein-DNA complex subunit organization (GO:0071824)
SmaSNP_306	3′ UTR	behavioral response to stimulus (GO:0007610)
SmaSNP_307	Synonymous	reproduction (GO:0000003)
SmaSNP_308	Non synonymous	Not found
SmaSNP_309	Synonymous	testosterone secretion (GO:0035936)
SmaSNP_310	3′ UTR	dynein-driven meiotic oscillatory nuclear movement (GO:0030989)
SmaSNP_311	3′ UTR	suppression by virus of host tapasin activity (GO:0039591)
SmaSNP_312	5′ UTR	Not found
SmaSNP_314	Synonymous	regulation of macrophage inflammatory protein 1 alpha production (GO:0071640)
SmaSNP_315	3′ UTR	menopause (GO:0042697)
SmaSNP_316	3′ UTR	T-helper 1 cell activation (GO:0035711)
SmaSNP_317	5′ UTR	Not found
SNP Name	SNP location/effect	GO term
SmaSNP_318	Unknown	Not found
SmaSNP_319	5′ UTR	phosphorylation (GO:0016310)
SmaSNP_320	Unknown	Not found
SmaSNP_321	3′ UTR	Golgi to plasma membrane protein transport (GO:0043001)
SmaSNP_322	3′ UTR	regulation of ATP citrate synthase activity (GO:2000983)
SmaSNP_323	3′ UTR	regulation of G-protein beta subunit-mediated signal transduction in response to host (GO:0075162)
SmaSNP_324	Non synonymous	cytokinesis, actomyosin contractile ring assembly (GO:0000915)
SmaSNP_325	Non synonymous	tear secretion (GO:0070075)
SmaSNP_326	5′ UTR	Not found
SmaSNP_328	Unknown	Not found
SmaSNP_329	5′ UTR	regulation of type II hypersensitivity (GO:0002892)
SmaSNP_330	Unknown	Not found
SmaSNP_332	5′ UTR	phosphorylation (GO:0016310)
SmaSNP_333	5′ UTR	Not found
SmaSNP_334	5′ UTR	Not found
SmaSNP_337	3′ UTR	juvenile-hormone esterase activity (GO:0004453)
SmaSNP_338	3′ UTR	inflammatory response to antigenic stimulus (GO:0002437)
SmaSNP_339	Synonymous	T-helper 1 cell activation (GO:0035711)
SmaSNP_340	Synonymous	regulation of G-protein coupled receptor protein signaling pathway (GO:0008277)
SmaSNP_341	Synonymous	sperm entry (GO:0035037)
SmaSNP_342	Unknown	Not found
SmaSNP_343	Synonymous	collagen metabolic process (GO:0032963)
SmaSNP_346	3′ UTR	chromatin silencing at silent mating-type cassette (GO:0030466)
SmaSNP_347	3′ UTR	nucleoside oxidase activity (GO:0033715)
SmaSNP_348	5′ UTR	retinoic acid receptor signaling pathway (GO:0048384)
SmaSNP_349	3′ UTR	T-helper 1 cell activation (GO:0035711)

**Table 3:** Description of two transcriptome 454-pyrosequencing runs of turbot.

	Inmune 1	Hypothalamic pituitary-gonad axis 2
**Samples**		
Number of individuals	52	30
Origin	Commercial fish farm	Commercial fish farm
**Data**		
Number of reads	915,782	1,191,866
Total megabases (Mb)	291.04	341.20
Average read length	317.8	286.0
**Assembly**		
Number of contigs	55,504	65,472
Mean length (bp)	671.3	625.9
Average contig coverage	4.4	4.6

1 From Pereiro *et al.* [10];

2 From Rivas *et al.* [11].

## Supplementary Files

Supplementary File 1 Supplementary Table (XLSX, 26 KB)

## References

[1] APROMAR (2012). La Acuicultura Marina en Espana.

[2] Bouza C., Presa P., Castro J., Sanchez L., Martinez P. (2002). Allozyme and microsatellite diversity in natural and domestic populations of turbot (*Scophthalmus maximus*) in comparison with other Pleuronectiformes. Can. J. Fish. Aquat. Sci.

[3] Castro J., Bouza C., Sanchez L., Cal R.M., Piferrer F., Martinez P. (2003). Gynogenesis assessment using microsatellite genetic markers in turbot (*Scophthalmus maximus*). Mar. Biotechnol.

[4] Castro J., Bouza C., Presa P., Pino-Querido A., Riaza A., Ferreiro I., Sanchez L., Martinez P. (2004). Potential sources of error in parentage assessment of turbot (*Scophthalmus maximus*) using microsatellite loci. Aquaculture.

[5] Sanchez-Molano E., Cerna A., Toro M.A., Bouza C., Hermida M., Pardo B.G., Cabaleiro S., Fernandez J., Martinez P. (2011). Detection of growth-related QTL in turbot (*Scophthalmus maximus*). BMC Genomics.

[6] Rodriguez-Ramilo S.T., Toro M.A., Bouza C., Hermida M., Pardo B.G., Cabaleiro S., Martinez P., Fernandez J. (2011). QTL detection for *Aeromonas salmonicida* resistance related traits in turbot (*Scophthalmus maximus*). BMC Genomics.

[7] Bouza C., Hermida M., Pardo B.G., Vera M., Fernandez C., de la Herran R., Navajas-Perez R., Alvarez-Dios J.A., Gomez-Tato A., Martinez P. (2012). An Expressed Sequence Tag (EST)-enriched genetic map of turbot (*Scophthalmus maximus*): A useful framework for comparative genomics across model and farmed teleosts. BMC Genetics.

[8] Vilas R., Bouza C., Vera M., Millan A., Martinez P. (2010). Variation in anonymous and EST-microsatellites suggests adaptive population divergence in turbot. Mar. Ecology-Prog. Ser.

[9] Pardo B.G., Fernandez C., Millan A., Bouza C., Vazquez-Lopez A., Vera M., Alvarez-Dios J.A., Calaza M., Gomez-Tato A., Vazquez M. (2008). Expressed sequence tags (ESTs) from immune tissues of turbot (*Scophthalmus maximus*) challenged with pathogens. BMC Vet. Res.

[10] Pereiro P., Balseiro P., Romero A., Dios S., Forn-Cuni G., Fuste B., Planas J.V., Beltran S., Novoa B., Figueras A. (2012). High-Throughput sequence analysis of turbot (*Scophthalmus maximus*) transcriptome using 454-pyrosequencing for the discovery of antiviral immune genes. PLoS One.

[11] Ribas L., Pardo B.G., Fernandez C., Alvarez-Dios J.A., Gomez-Tato A., Quiroga M.I., Planas J., Sitja-Bobadilla A., Martinez P., Piferrer F (2013). A combined strategy involving Sanger and 454 pyrosequencing increases genomic resources to aid in the management of reproduction; disease control and genetic selection in the turbot (*Scophthalmus maximus*). BMC Genomics.

[12] Metzker M.L. (2010). Applications of next-generation sequencing: Sequencing technologies—The next generation. Nat. Rev. Genetics.

[13] Voelkerding K.V., Dames S.A., Durtschi J.D. (2009). Next-Generation sequencing: From basic research to diagnostics. Clin. Chem.

[14] Adams M.D., Kelley J.M., Gocayne J.D., Dubnick M., Polymeropoulos M.H., Xiao H., Merril C.R., Wu A., Olde B., Moreno R.F. (1991). Complementary DNA sequencing— Expressed sequence tags and human genome project. Science.

[15] Marra M.A., Hillier L., Waterston R.H. (1998). Expressed sequence tags-ESTablishing bridges between genomes. Trends Genetics.

[16] Liu Z.J., Li P., Kocabas A., Karsi A., Ju Z.L. (2001). Microsatellite-containing genes from the channel catfish brain: Evidence of trinucleotide repeat expansion in the coding region of nucleotide excision repair gene RAD23B. Biochem. Biophys. Res. Commu.

[17] Serapion J., Kucuktas H., Feng J.A., Liu Z.J. (2004). Bioinformatic mining of type I microsatellites from expressed sequence tags of channel catfish (*Ictalurus punctatus*). Mar. Biotechnol.

[18] He C., Chen L., Simmons M., Li P., Kim S., Liu Z.J. (2003). Putative SNP discovery in interspecific hybrids of catfish by comparative EST analysis. Anim. Genetics.

[19] Bouza C., Hermida M., Pardo B.G., Fernandez C., Fortes G.G., Castro J., Sanchez L., Presa P., Perez M., Sanjuan A. (2007). A microsatellite genetic map of the turbot (*Scophthalmus maximus*). Genetics.

[20] Moen T., Hayes B., Nilsen F., Delghandi M., Fjalestad K.T., Fevolden S.E., Berg P.R., Lien S. (2008). Identification and characterisation of novel SNP markers in Atlantic cod: Evidence for directional selection. BMC Genetics.

[21] Morin P.A., Luikart G., Wayne R.K., Grp S.N.P.W. (2004). SNPs in ecology; evolution and conservation. Trends Ecol. Evol.

[22] Ferber S., Reusch T.B.H., Stam W.T., Olsen J.L. (2008). Characterization of single nucleotide polymorphism markers for eelgrass (*Zostera marina*). Mol. Ecol. Resour.

[23] Stapley J., Reger J., Feulner P.G.D., Smadja C., Galindo J., Ekblom R., Bennison C., Ball A.D., Beckerman A.P., Slate J. (2010). Adaptation genomics: The next generation. Trends Ecol. Evol.

[24] Kim S., Misra A. (2007). SNP genotyping: Technologies and biomedical applications. Ann. Rev. Biomed. Eng.

[25] Bester A.E., Roodt-Wilding R., Whitaker H.A. (2008). Discovery and evaluation of single nucleotide polymorphisms (SNPs) for *Haliotis midae*: A targeted EST approach. Anim. Genetics.

[26] Sachidanandam R., Weissman D., Schmidt S.C., Kakol J.M., Stein L.D., Marth G., Sherry S., Mullikin J.C., Mortimore B.J., Willey D.L. (2001). A map of human genome sequence variation containing 1.42 million single nucleotide polymorphisms. Nature.

[27] Reich D.E., Gabriel S.B., Altshuler D. (2003). Quality and completeness of SNP databases. Nat. Genetics.

[28] Brumfield R.T., Beerli P., Nickerson D.A., Edwards S.V. (2003). The utility of single nucleotide polymorphisms in inferences of population history. Trends Ecol. Evol.

[29] Vera M., Alvarez-Dios J.A., Millan A., Pardo B.G., Bouza C., Hermida M., Fernandez C., de la Herran R., Molina-Luzon M.J., Martinez P. (2011). Validation of single nucleotide polymorphism (SNP) markers from an immune Expressed Sequence Tag (EST) turbot; *Scophthalmus maximus*; database. Aquaculture.

[30] Stickney H.L., Schmutz J., Woods I.G., Holtzer C.C., Dickson M.C., Kelly P.D., Myers R.M., Talbot W.S. (2002). Rapid mapping of zebrafish mutations with SNPs and oligonucleotide microarrays. Genome Res.

[31] Cenadelli S., Maran V., Bongioni G., Fusetti L., Parma P., Aleandri R. (2007). Identification of nuclear SNPs in gilthead seabream. J. Fish. Biol.

[32] Hayes B., Laerdahl J.K., Lien S., Moen T., Berg P., Hindar K., Davidson W.S., Koop B.F., Adzhubei A., Hoyheim B. (2007). An extensive resource of single nucleotide polymorphism markers associated with Atlantic salmon (*Salmo salar*) expressed sequences. Aquaculture.

[33] Wang S.L., Sha Z.X., Sonstegard T.S., Liu H., Xu P., Somridhivej B., Peatman E., Kucuktas H., Liu Z.J. (2008). Quality assessment parameters for EST-derived SNPs from catfish. BMC Genomics.

[34] Hubert S., Bussey J.T., Higgins B., Curtis B.A., Bowman S. (2009). Development of single nucleotide polymorphism markers for Atlantic cod (*Gadus morhua*) using expressed sequences. Aquaculture.

[35] Hubert S., Higgins B., Borza T., Bowman S. (2010). Development of a SNP resource and a genetic linkage map for Atlantic cod (*Gadus morhua*). BMC Genomics.

[36] Sauvage C., Bierne N., Lapegue S., Boudry P. (2007). Single nucleotide polymorphisms and their relationship to codon usage bias in the Pacific oyster *Crassostrea gigas*. Gene.

[37] Vera M., Pardo B.G., Pino-Querido A., Alvarez-Dios J.A., Fuentes J., Martinez P. (2010). Characterization of single-nucleotide polymorphism markers in the Mediterranean mussel; *Mytilus galloprovincialis*. Aquac. Res.

[38] Zhang L.S., Guo X.M. (2010). Development and validation of single nucleotide polymorphism markers in the eastern oyster *Crassostrea virginica* Gmelin by mining ESTs and resequencing. Aquaculture.

[39] Du Z.Q., Ciobanu D.C., Onteru S.K., Gorbach D., Mileham A.J., Jaramillo G., Rothschild M.F. (2010). A gene-based SNP linkage map for pacific white shrimp; *Litopenaeus vannamei*. Anim. Genetics.

[40] Gorbach D.M., Hu Z.L., Du Z.Q., Rothschild M.F. (2010). Mining ESTs to determine the usefulness of SNPs across shrimp species. Anim. Biotechnol.

[41] Lepoittevin C., Frigerio J.M., Garnier-Gere P., Salin F., Cervera M.T., Vornam B., Harvengt L., Plomion C. (2010). *In vitro vs. in silico* detected SNPs for the development of a genotyping array: What can we learn from a non-model species?. PLoS One.

[42] Zhu C., Cheng L., Tong J., Yu X. (2012). Development and characterization of new single nucleotide polymorphism markers from expressed sequence tags in common carp (*Cyprinus carpio*). Int. J. Mol. Sci.

[43] Roche Diagnostics GmbH (2009). cDNA Rapid Library Preparation Method Manual.

[44] Campbell N.R., Amish S.J., Pritchard V.L., McKelvey K.S., Young M.K., Schwartz M.K., Garza J.C., Luikart G., Narum S.R. (2012). Development and evaluation of 200 novel SNP assays for population genetic studies of westslope cutthroat trout and genetic identification of related taxa. Mol. Ecol. Resour.

[45] Koski L.B., Gray M.W., Lang B.F., Burger G. (2005). AutoFACT: An (Auto)under-barmatic (F)under-barunctional (A)under-barnnotation and (C)under-barlassification (T)under-barool. BMC Bioinforma.

[46] Kim H., Schmidt C.J., Decker K.S., Emara M.G. (2003). A double-screening method to identify reliable candidate non-synonymous SNPs from chicken EST data. Anim. Genetics.

[47] Wondji C.S., Hemingway J., Ranson H. (2007). Identification and analysis of single nucleotide polymorphisms (SNPs) in the mosquito *Anopheles funestus*; malaria vector. BMC Genomics.

[48] Chevreux B., Pfisterer T., Drescher B., Driesel A., Müller W.E.G., Wetter T., Suhai S. (2004). Using the miraEST assembler for reliable and automated mRNA transcript assembly and dtection in sequenced ESTs. Genome Res.

[49] Huang X., Madan A. (1999). CAP3: A DNA sequence assembly program. Genome Res.

[50] Ueno S., le Provost G., Leger V., Klopp C., Noirot C., Frigerio J.-M., Salin F., Salse J., Abrouk M., Murat F. (2010). Bioinformatic analysis of ESTs collected by Sanger and pyrosequencing methods for a keystone forest tree species: Oak. BMC Genomics.

[51] Tang J., Vosman B., Voorrips R.E., Linden C.G., van der Linden C.G., Leunissen J.A.M. (2006). QualitySNP: A pipeline for detecting single nucleotide polymorphisms and insertions/deletions in EST data from diploid and polyploid species. BMC Bioinforma.

[52] MySQL Home Page.

[53] M-View Home Page.

[54] BLAST Home Page.

[55] Sambrook J., Fritsch E.F., Maniatis T (1989). Molecular Cloning: A Laboratory Manual.

[56] Buetow K.H., Edmonson M., MacDonald R., Clifford R., Yip P., Kelley J., Little D.P., Strausberg R., Koester H., Cantor C.R. (2001). High-throughput development and characterization of a genomewide collection of gene-based single nucleotide polymorphism markers by chip-based matrix-assisted laser desorption/ionization time-of-flight mass spectrometry. Proc. Natl. Acad. Sci. USA.

[57] Oeth P., del Mistro G., Marnellos G., Shi T., van den Boom D. (2009). Qualitative and quantitative genotyping using single base primer extension coupled with matrix-assisted laser desorption/ionization time-of-flight mass spectrometry (MassARRAY). Methods Mol. Biol.

[58] Goudet J FSTAT, a program to estimate and test gene diversities and fixation indices (version 2.9.3).

[59] Raymond M., Rousset F. (1995). GENEPOP (Version 1.2)—Population genetics software for exact tests and ecumenicism. J. Hered.

[60] Rousset F. (2008). GENEPOP’007: A complete re-implementation of the GENEPOP software for Windows and Linux. Mol. Ecol. Resour.

[61] Louis E.J., Dempster E.R. (1987). An exact test for Hardy-Weinberg and multiple alleles. Biometrics.

[62] Rice W.R. (1989). Analyzing tables of statistical tests. Evolution.

[63] ORF Finder Home Page.

[64] Thompson J.D., Higgins D.G., Gibson T.J. (1994). CLUSTAL W improving the sensitivity of progressive multiple sequence alignment through sequence weighting; position-specific gap penalties and weight matrix choice. Nucl. Acids Res.

[65] Hall T.A. (1999). BioEdit: A user-friendly biological sequence alignment editor and analysis program fro Windows 95/98/NT. Nucl. Acids Symp. Ser.

[66] QuickGO Home Page.

[67] AmiGO Home Page.

